# N-Acetylcysteine prevents congenital heart defects induced by pregestational diabetes

**DOI:** 10.1186/1475-2840-13-46

**Published:** 2014-02-18

**Authors:** Hoda Moazzen, Xiangru Lu, Noelle L Ma, Thomas J Velenosi, Brad L Urquhart, Lambertus J Wisse, Adriana C Gittenberger-de Groot, Qingping Feng

**Affiliations:** 1 Department of Physiology and Pharmacology, University of Western Ontario, London, Ontario N6A 5C1, Canada; 2 Department of Medicine, London, Ontario, Canada; 3 Lawson Health Research Institute, London, Ontario, Canada; 4 Department of Anatomy and Embryology, Leiden University Medical Center, Leiden, The Netherlands; 5 Department of Cardiology, Leiden University Medical Center, Leiden, The Netherlands

**Keywords:** Pregestational diabetes, Congenital heart defects, N-acetylcysteine, Glutathione, Reactive oxygen species

## Abstract

**Background:**

Pregestational diabetes is a major risk factor of congenital heart defects (CHDs). Glutathione is depleted and reactive oxygen species (ROS) production is elevated in diabetes. In the present study, we aimed to examine whether treatment with N-acetylcysteine (NAC), which increases glutathione synthesis and inhibits ROS production, prevents CHDs induced by pregestational diabetes.

**Methods:**

Female mice were treated with streptozotocin (STZ) to induce pregestational diabetes prior to breeding with normal males to produce offspring. Some diabetic mice were treated with N-acetylcysteine (NAC) in drinking water from E0.5 to the end of gestation or harvesting of the embryos. CHDs were identified by histology. ROS levels, cell proliferation and gene expression in the fetal heart were analyzed.

**Results:**

Our data show that pregestational diabetes resulted in CHDs in 58% of the offspring, including ventricular septal defect (VSD), atrial septal defect (ASD), atrioventricular septal defects (AVSD), transposition of great arteries (TGA), double outlet right ventricle (DORV) and tetralogy of Fallot (TOF). Treatment with NAC in drinking water in pregestational diabetic mice completely eliminated the incidence of AVSD, TGA, TOF and significantly diminished the incidence of ASD and VSD. Furthermore, pregestational diabetes increased ROS, impaired cell proliferation, and altered *Gata4*, *Gata5* and *Vegf-a* expression in the fetal heart of diabetic offspring, which were all prevented by NAC treatment.

**Conclusions:**

Treatment with NAC increases GSH levels, decreases ROS levels in the fetal heart and prevents the development of CHDs in the offspring of pregestational diabetes. Our study suggests that NAC may have therapeutic potential in the prevention of CHDs induced by pregestational diabetes.

## Introduction

Congenital heart defects (CHDs) are the most common birth defects affecting up to 5% of live births in the general population [1]. Pregestational diabetes mellitus, either type 1 or type 2, increases the risk of CHDs in infants by 3–5 fold compared to non-diabetic pregnancies [2-6]. With an increase in the number of young adults having diabetes mellitus [7,8], the incidence of pregestational diabetes and CHDs caused by maternal diabetes may further increase, with significant social and economic consequences.

Although factors responsible for the high incidence of CHDs in pregestational diabetes are still not fully understood, evidence suggests that oxidative stress may play a role [9,10]. For example, the antioxidant capacity of the developing embryo is limited [11,12], and reactive oxygen species (ROS) production is exacerbated as the expression and activities of major ROS scavenging enzymes including superoxide dismutase and glutathione peroxidase are decreased during maternal diabetes [13-15]. In addition, maternal hyperglycemia diminishes the level of an important intracellular antioxidant, glutathione (GSH) [16,17], which places the developing embryo in an extremely vulnerable state to oxidative stress.

N-Acetylcysteine (NAC) is a thiol-containing antioxidant agent and can cross the placenta [18]. The main biological effect of NAC as a precursor of cysteine is to replenishing cellular GSH levels and to preserve the thiol redox status. Additionally, NAC also reacts with hydroxyl radical (OH), nitrogen dioxide (NO_2_) and thiyl radicals to reduce oxidative stress [19]. Furthermore, NAC treatment *in vitro* and *in ovo* diminishes high glucose-induced developmental defects in mouse and chicken embryos [10,20]. In the present study, we hypothesized that NAC treatment in diabetic mice during gestation diminishes ROS production and prevents the development of CHDs in their offspring. To test this hypothesis, a pregestational diabetes mouse model was established to closely simulate CHDs in patients with pregestational diabetes. We demonstrated that NAC treatment in pregestational diabetic mice decreased ROS levels and improved cell proliferation during embryonic heart development, and prevented CHDs in the diabetic offspring.

## Methods

### Animals

C57BL/6 wild type mice were purchased from Jackson Laboratory (Bar Harbor, Maine). A breeding program was implemented to generate fetal and postnatal mice. Animals in this study were handled in accordance with the *Guide for the Care and Use of Laboratory Animals*, published by the U.S. National Institutes of Health (NIH publ. no. 85–23, revised 1996). Use of animals was approved by the Animal Use Subcommittee at the University of Western Ontario, Canada.

### Induction of diabetes mellitus and N-acetylcysteine treatment

Eight weeks old C57BL/6 female mice were treated with streptozotocin (STZ, 80 mg/kg body weight, IP, Sigma, Canada) for 3 consecutive days. Mice treated with saline served as controls. Non-fasting blood glucose levels were determined one week after STZ injection using a glucose meter (OneTouch Ultra2, LifeScan, Canada, Burnaby, BC, Canada). Mice with blood glucose levels higher than 11 mmol/L were bred to normal adult males. Mating was verified by observation of a vaginal plug, which was counted as day E0.5 of pregnancy. A subset of control and diabetic mice received 4 mg/ml N-acetylcysteine (NAC, 1 g/kg body weight/day) in drinking water [21] from E0.5 to the end of gestation or harvesting of the embryos. Non-fasting blood glucose levels were monitored in all groups during gestation.

### Histological analysis

Heart morphology was analyzed in postnatal day 0 (P0) mice and cell proliferation was analyzed by phospho-histon H3 (pHH3) staining in E12.5 hearts. Briefly, the mouse thorax was fixed in 4% paraformaldehyde overnight, dehydrated in ethanol, embedded in paraffin and serially sectioned into 5-μm sections. Heart sections were stained with hematoxylin/eosin (H/E) and images were captured using a light microscope (Observer D1, Zeiss, Germany). Images were taken on every 25 μm of the heart and the three-dimensional visualization of heart structures was reconstructed using AMIRA® program. To analyze cell proliferation and apoptosis, heart sections were immunostained using anti-pHH3 (phospho S10) antibody (Abcam) and anti-cleaved claspase-3 antibody (Cell Signaling), respectively, followed by incubation with biotinylated goat anti-rabbit IgG (Vector Laboratories, Burlingame, CA, USA). Signals were visualized by 3-3′di-aminobenzidin tetrahydrochloride (Sigma-Aldrich Chemie, St. Louis, MO, USA). Counterstaining was performed with modified Mayer’s hematoxylin (Thermo Scientific, Waltham, MA, USA). The number of pHH3^+^ cells from at least 3 individual heart sections per sample was quantified and normalized to areas of the myocardium.

### Analysis of superoxide levels

Embryonic heart tissues were harvested at E12.5 in all four groups. Frozen samples were cut into 10-μm sections using a cryostat (CM1950, Leica, Germany). Superoxide levels were assessed by incubation of heart sections with 2 μM dihydroethidium (DHE) (Invitrogen Life Technologies, Burlington, Canada) for 30 minutes in a humidified and light protected chamber in room air at 37°C [22]. DHE fluorescence signals were detected using a fluorescence microscope (Observer D1, Zeiss, Germany). For analysis of superoxide levels, 5–8 images of each heart sample were captured using fixed exposure time for all groups. The intensity of fluorescence signals per myocardial area was quantified using AxioVision software. A limitation of this assay is that the oxygen level was not adjusted to that of the embryonic hearts *in vivo*[23].

### Real-time RT-PCR analysis

Total RNA was extracted from individual E11.5 fetal hearts using RNeasy Mini kit (Qiagen, Burlington, ON, Canada) as per manufacturer’s instructions. One hundred nanograms of total RNA were used to synthesize cDNA using M-MLV reverse transcriptase. Real-time PCR was conducted using EvaGreen qPCR MasterMix (Applied Biological Materials, Vancouver, BC, Canada). Specific primers were designed for *Nkx2.5*, *Gata4*, *Gata5*, *Tbx5*, *Tgf-β1*, *Vegf-a*, *Mef2c*, *cyclin D1* and *Bmp4* (Table 1). Samples were amplified for 35 cycles using Eppendorf Realplex (Eppendorf, Hamburg, Germany). Values were normalized with 28S ribosomal RNA. The mRNA levels in relation to 28S rRNA were determined using a comparative C_T_ method [24].

**Table 1:** Primer sequences for real-time PCR analysis

**Gene**	**Accession no.**	**Product size**	**Primer sequence (5′→3′)**
*Nkx2.5*	NM_008700.2	162	F: GACAGCGGCAGGACCAGACT
			R: CGTTGTAGCCATAGGCATTG
*Vegfa*	NM_001025257.3	194	F: GATTGAGACCCTGGTGGACAT
			R: TCTCCTATGTGCTGGCTTTGG
*Gata4*	NM_008092.3	134	F: GCCTGCGATGTCTGAGTGAC
			R: CACTATGGGCACAGCAGCTC
*Gata5*	NM_008093.2	167	F: ACCCCACAACCTACCCAGCA
			R: GCCCTCACCAGGGAACTCCT
*Bmp4*	NM_007554.2	250	F: GTTATGAAGCCCCCAGCAGA
			R: CCCAATCTCCACTCCCTTGA
*Tbx5*	NM_011537.3	103	F: AGGAGCACAGTGAGGCACAA
			R: GGGCCAGAGACACCATTCTC
*Tgf-β1*	NM_011577.1	120	F: GCCCGAAGCGGACTACTATG
			R: CACTGCTTCCCGAATGTCTG
*28S*	NR_003279.1	178	F: GGGCCACTTTTGGTAAGCAG
			R: TTGATTCGGCAGGTGAGTTG
*Cyclin D1*	NM_007631.2	135	F: CTGACACCAATCTCCTCAACG
			R: CTCACAGACCTCCAGCATCCA
*Mef2c*	NM_001170537.1	405	F: CACCGAGTACAACGAGCCGCA
			R: CTGGTGCCTGCACCGGATGTC

F: forward primer, R: reverse primer.

### Glutathione levels in fetal hearts

Briefly, E14.5 fetal hearts were washed in PBS and snap frozen in liquid nitrogen. Heart samples were homogenized in 6% sulfosalicylic acid and 1 mM EDTA then centrifuged at 8,000 g for 5 minutes at 4°C. Total and reduced glutathione were assessed using a modified ultra-performance liquid chromatography (UPLC) method [25,26]. N-isoamyl alcohol was added to 50 μL of supernatant fraction of all samples. To determine total glutathione, thiols were reduced with NaBH_4_ followed by the addition of HCl to adjust the pH to approximately 8.0, and then derivatized by the addition of 25 mM monobromobimane. To determine reduced glutathione, the pH of the sample was raised to approximately 8.0 with NaOH and the samples were immediately derivatized as described above. Following derivatization, the pH of all samples was decreased to approximately 4.0 with glacial acetic acid and 5 μL was injected onto a Kinetex C18 column (50 × 2.1 mm, 1.7 μm particle, Phenomenex, Torrance, CA) which was maintained at 40°C in a Waters AQUITY UPLC™ H-Class System. The mobile phase consisted 5% acetonitrile and 95% 5 mM KH_2_PO_4_ with 0.1% triethylamine, pH 4.0. The derivatized glutathione was detected by a Waters ACQUITY UPLC® fluorescence detector with the excitation set to 390 nm and the emission set to 480 nm.

### Statistical analysis

Data are presented as means ± SEM. Statistical analysis was performed using two-way analysis of variance (ANOVA) followed by Bonferroni post test. The incidence of congenital malformations was analyzed by Chi-square test. *P* < 0.05 was considered statistically significant.

## Results

### Effects of NAC on maternal blood glucose levels, litter size and mortality at birth

**Figure 1:**
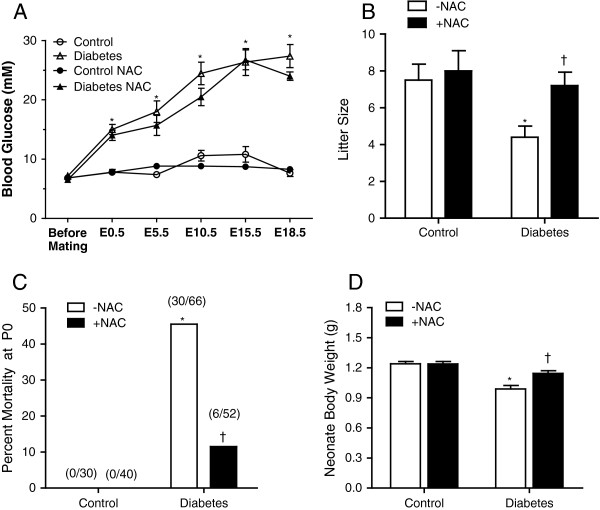
**Blood glucose levels of pregnant mice, litter size, mortality and body weight of neonates at P0. (A)** Non-fasting blood glucose levels before mating (basal), and E0.5 to E18.5 after pregnancy in STZ-treated and control female mice with and without NAC treatment (*n* = 7-10 mice per group). **(B)** The offspring litter size. **(C)** Mortality of neonates at birth. The numbers in brackets indicate the number of death to total. **(D)** Body weight of the offspring at birth (*n* = 27-29 per group). *P < 0.001 vs. untreated control, †*P* < 0.001 vs. untreated diabetes. Data are means ± SEM.

One week after STZ injection, female mice with blood glucose levels higher than 11 mM were set up to breed with normal males. Diabetic mice had significantly higher blood glucose levels at the time of vaginal plugging (E0.5) compared to controls (*P <* 0.001, Figure 1A). Additionally, time to vaginal plugging that lead to successful pregnancy was 10 times longer in the diabetic compared to control mice (26.7±6.1 vs. 2.7±0.9 days, *P <* 0.01), indicating decreased fertility rate in diabetic females. From E0.5 to E18.5 of gestation, blood glucose levels of diabetic mice were progressively increased but not significantly altered by NAC treatment (Figure 1A). Average litter size of diabetic neonates at P0 was significantly less than controls (Figure 1B). Additionally, diabetic neonates had 46% mortality rate at birth (*P* < 0.001, Figure 1C). Administration of NAC in diabetic dams improved litter size of the offspring (*P* < 0.05) and diminished their mortality at birth to 11.5% (*P* < 0.001). The body weight of neonates born to diabetic mice was significantly lower compared to controls at P0 (*P <* 0.001, Figure 1D). NAC treatment did not affect body weight of neonates in control mice, but significantly improved the body weight of the diabetic offspring (*P <* 0.001, Figure 1D).

### Effects of NAC on incidence of CHDs in diabetic offspring

**Figure 2:**
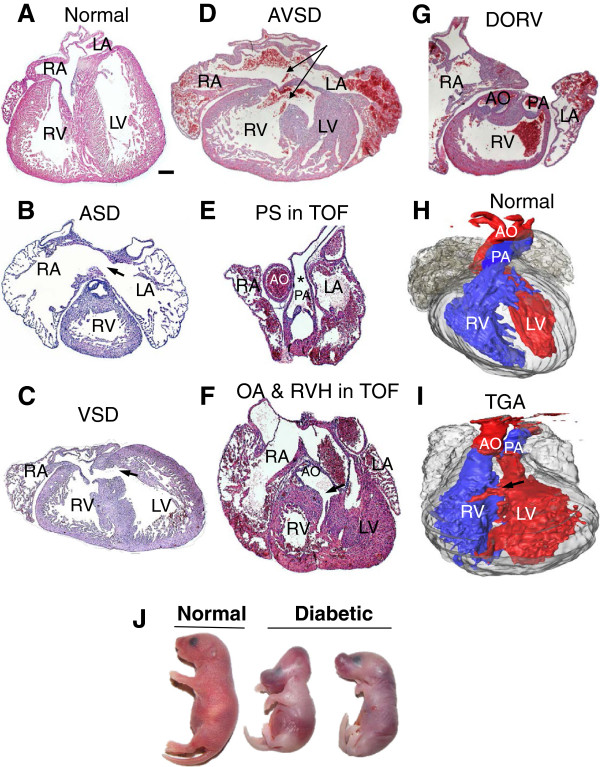
**Congenital defects in the diabetic offspring at P0. (A)** A normal heart of control offspring. The offspring of mice with pregestational diabetes show **(B)** atrial septal defect (ASD), **(C)** ventricular septal defect (VSD), **(D)** atrioventricular septal defect (AVSD), **(E and F)** tetralogy of Fallot (TOF). **(E)** *pulmonary stenosis (PS), **(F)** VSD, overriding aorta (OA) marked by an arrow, and RV hypertrophy (RVH), **(G)** double outlet right ventricle (DORV), and **(I)** transposition of great arteries (TGA). Panels **(H)** and **(I)** are 3D-reconstructed images of a normal heart and a TGA, respectively. In panel **(H)** the aorta (red) is connected to the right ventricle (RV) while the pulmonary artery (PA in blue) is connected to LV. This defect is associated with a VSD. **(J)** Craniofacial defect in diabetic offspring. RA: right atria, LA: left atria, LV: left ventricle. Arrows indicate ASD or VSD. Scale bar is 200 μm.

Pregestational diabetes resulted in 58.1% CHDs in the offspring (Table 2). The majority of the defects were malformations of the septum with 30.6% atrial septal defect (ASD, Figure 2B) and 40.3% ventricular septal defect (VSD, Figure 2C). In addition, 6.5% of diabetic offspring showed atrioventricular septal defect (AVSD, Figure 2D). Defects in the outflow tract included 12.9% double outlet right ventricle (DORV, Figure 2G) and 6.5% transposition of great arteries (TGA, Figure 2I). Furthermore, 4.8% of diabetic offspring showed tetralogy of fallot (TOF) with pulmonary stenosis (Figure 2E), overriding aorta and VSD associated with right ventricle hypertrophy (Figure 2F). Treatment with NAC in diabetic mice during gestation significantly reduced the incidence of CHDs to 16.3% (Table 2). Specifically, NAC treatment decreased incident of ASD and VSD to 13.9% and 11.6%, respectively. Also, outflow tract formation and remodeling were improved as the rate of DORV was reduced to 6.9% while AVSD, TGA and TOF were fully rescued by NAC treatment in the diabetic mice (Table 2). Craniofacial defects were also observed in diabetic embryos (4.8%, Figure 2J). However, none of the controls or NAC treated groups showed any craniofacial defects.

**Table 2:** The rate of congenital heart defects in the offspring of diabetic and control females with and without N-acetylcysteine (NAC) treatment

**Total N/litters**	**Control 30/4**		**Diabetes 62/15**		**Control NAC 30/4**		**Diabetes NAC 43/7**	
	**n**	**%**	**n**	**%**	**n**	**%**	**n**	**%**
Normal	30	100	26	41.9**	27	90	36	83.7††
Abnormal	0	0	36	58.1**	3	10	7	16.3††
ASD	0	0	19	30.6**	2	6.7	6	13.9†
VSD	0	0	25	40.3**	1	3.3	5	11.6††
AVSD	0	0	4	6.5	0	0	0	0
TGA	0	0	4	6.5	0	0	0	0
DORV	0	0	8	12.9*	0	0	3	6.9
TOF	0	0	3	4.8	0	0	0	0

Data were analyzed by Chi-square test. *P < 0.05, **P < 0.001 vs. untreated control, †*P* < 0.05, ††*P* < 0.001 vs. untreated diabetes. ASD, atrial septal defect; VSD, ventricular septal defect; AVSD, atrioventricular septal defect; TGA, transposition of great arteries; DORV, double outlet right ventricle; TOF, Tetralogy of Fallot.

### Effects of NAC on glutathione and ROS levels in the heart of diabetic offspring

**Figure 3:**
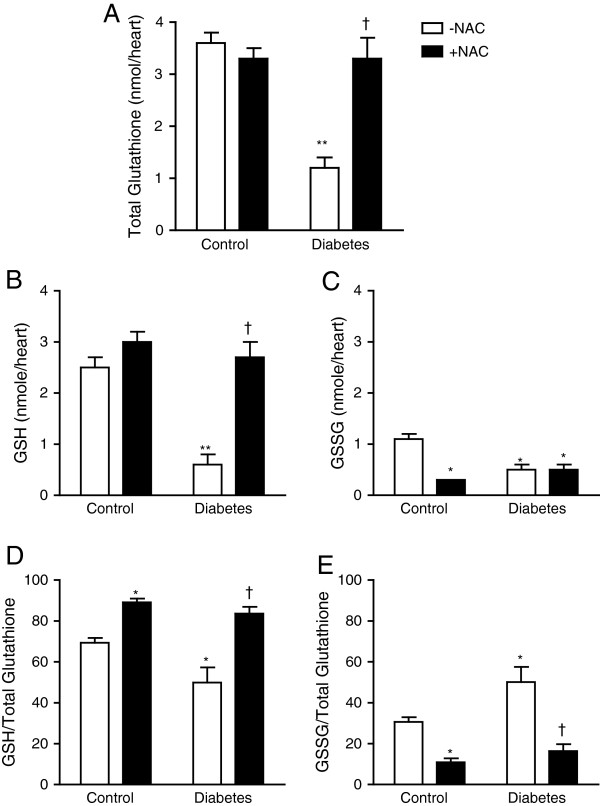
**Measurement of intracellular glutathione levels in fetal hearts at E14.5. (A)** Total glutathione levels, **(B)** Reduced glutathione (GSH) levels, **(C)** Oxidized glutathione (GSSG) levels, **(D)** GSH to total glutathione ratio, and **(E)** GSSG to total glutathione ratio. Data are means ± SEM, *n =* 7–9 samples per group. *P < 0.05, **P < 0.01 vs. untreated control, †*P* < 0.001 vs. untreated diabetes.

**Figure 4:**
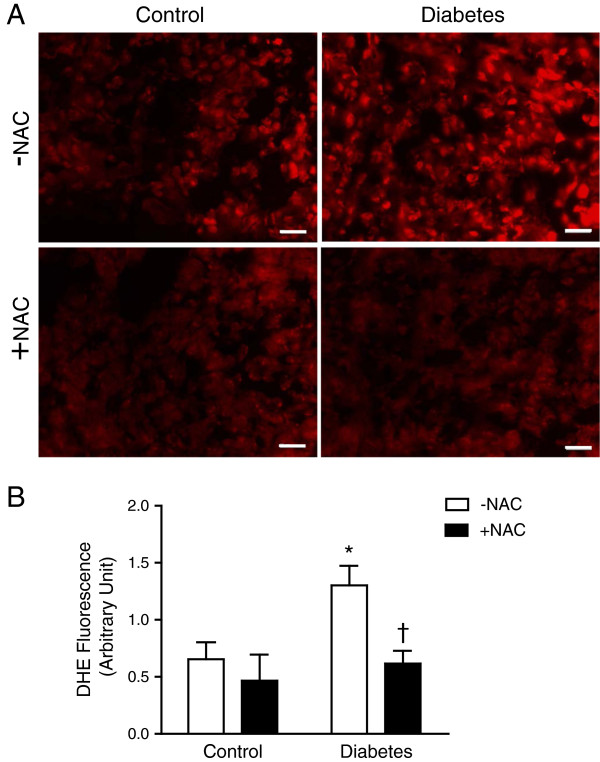
**Analysis of superoxide levels in fetal heart at E12.5 using dihydroethidium (DHE) as a probe. (A)** Representative images of DHE staining in the LV myocardium of fetal hearts. **(B)** Quantification of DHE fluorescence intensity. Data are means ± SEM, *n* = 5–6 samples per group. *P < 0.05 vs. untreated control, †*P* < 0.05 vs. untreated diabetes. Scale bar is 20 μm.

Fetal glutathione levels were measured in E14.5 hearts. Diabetic offspring demonstrated significant reductions in total glutathione, GSH and GSSG levels compared to non-diabetic controls (*P* < 0.01, Figure 3A-C). The GSH/total glutathione ratio was decreased while GSSG/total glutathione ratio was increased in diabetic fetal hearts (*P* < 0.05, Figure 3D and E). NAC treatment completely restored total glutathione and GSH levels in the diabetic offspring (*P* < 0.001, Figure 3A and B), leading to increased GSH/total glutathione ratio and decreased GSSG/total glutathione ratio (*P* < 0.01, Figure 3D-E). NAC treatment in non-diabetic females during gestation had no effect on total glutathione levels but decreased GSSG levels, and consequently GSH/total glutathione ratio was increased (*P* < 0.05, Figure 3D). To examine the effects of NAC on ROS levels, dihydroethidium (DHE) was employed as a probe to assess superoxide generation in fetal hearts at E12.5. Elevated DHE fluorescence reading in the fetal heart of diabetic offspring indicated excess superoxide levels, which was significantly inhibited by NAC treatment (*P* < 0.05, Figure 4).

### Effects of NAC on cell proliferation and apoptosis in the heart of diabetic offspring

**Figure 5:**
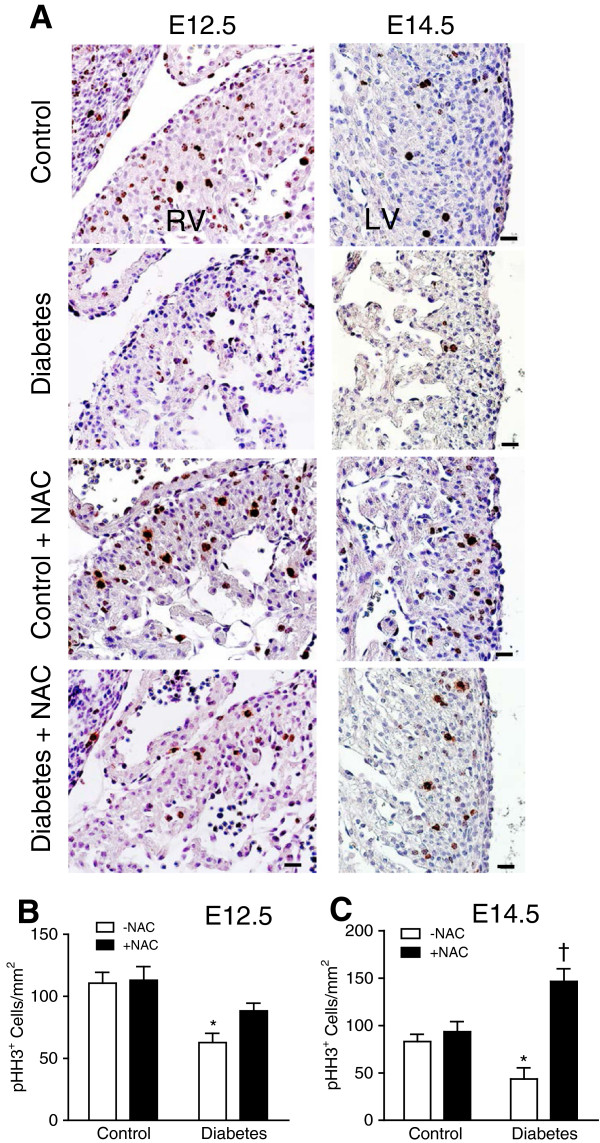
**Cell proliferation in the fetal ventricular myocardium. (A)** Representative histological heart sections immunostained for pHH3. pHH3^+^ staining (brown) is localized in the nucleus. **(B and C)** Quantification of pHH3^+^ cells in heart tissue sections, which include the right, left and septal myocardium. Data are means ± SEM, *n* = 6–8 per group *P < 0.05 vs. untreated control, †*P* < 0.001 vs. untreated diabetes. Scale bar is 50 μm.

**Figure 6:**
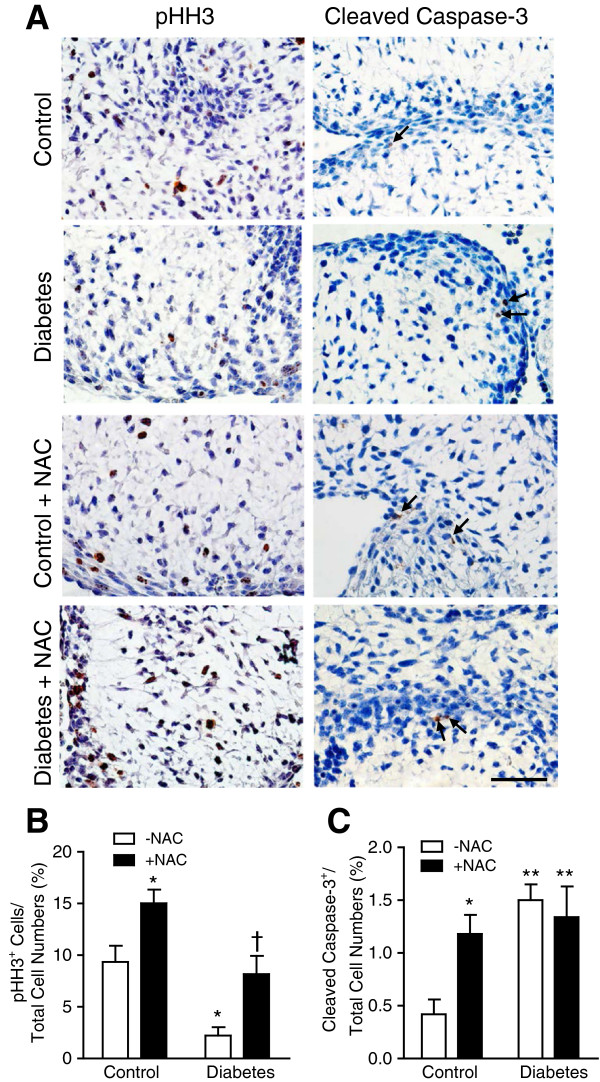
**Cell proliferation and apoptosis in the endocardial cushion at E12.5. (A)** Representative immunohistological staining of pHH3 (nucleus) and cleaved caspase-3. **(B and C)** Quantification of pHH3^+^ cells (cell proliferation) and cleaved caspase-3^+^ cells (apoptosis) in relation to total endocardial cushion cells. *P < 0.05, **P < 0.01 vs. untreated control, †*P* < 0.05 vs. untreated diabetes. Scale bar is 50 μm.

**Figure 7:**
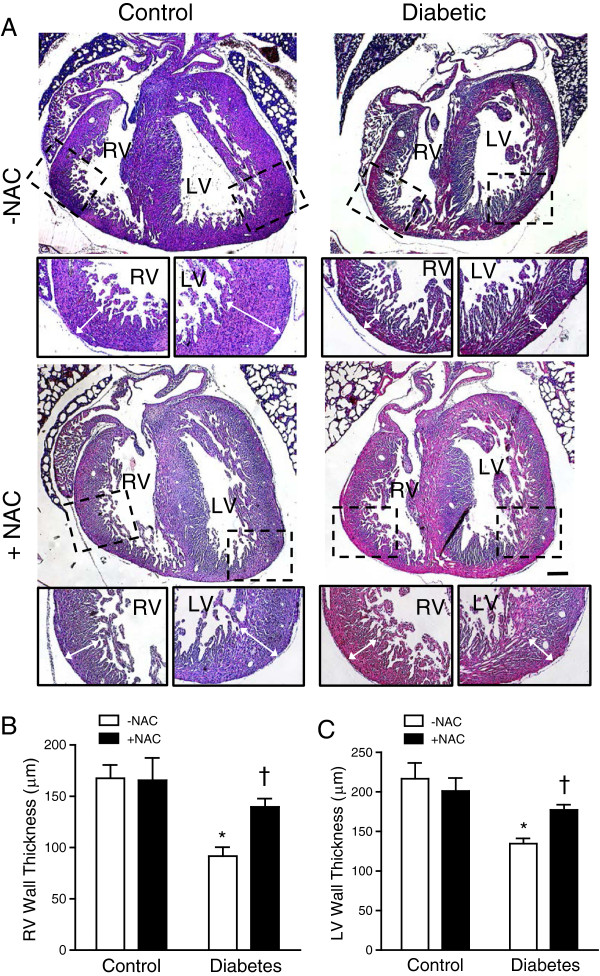
**Myocardial wall thickness at P0. (A)** Representative images of myocardial wall thickness. **(B and C)** Quantification of right and left ventricular wall thickness. *n* = 5-6 per group, *P < 0.01 vs. untreated control, †*P* < 0.05 vs. untreated diabetes. Scale bar is 200 μm.

Proper cell proliferation and apoptosis are essential for normal embryonic heart development [27,28]. Using phosphorylated histone H3 (pHH3) as a marker, we analyzed cell proliferation in fetal hearts at E12.5 and E14.5. The offspring of mice with pregestational diabetes showed a significant decrease in the number of myocardial proliferating cells at E12.5 and E14.5 (*P* < 0.05, Figure 5A-C). NAC treatment during gestation showed a trend but not a statistically significant increase in cell proliferation at E12.5. Notably, this effect was significant at E14.5 (*P* < 0.001, Figure 5C). In E12.5 endocardial cushion, cell proliferation was decreased in the diabetic offspring (Figure 6A, B). NAC treatment significantly increased cell proliferation in both controls and diabetic offspring (Figure 6B). We also assessed cell apoptosis through immunohistochemical analysis of cleaved caspase-3 (Figure 6A). Apoptosis in the endocardial cushion at E12.5 was significantly increased in the diabetic offspring compared to control embryos (*P* < 0.05, Figure 6A, C). NAC treatment had no significant effect on apoptosis in the diabetic offspring, but increased apoptosis in the endocardial cushion in control embryos (*P* < 0.05, Figure 6C). As a result of reduced cell proliferation, myocardial wall thickness was decreased in the diabetic offspring at P0 (*P* < 0.01, Figure 7A-C), which was rescued by NAC treatment (*P* < 0.05, Figure 7A-C).

### Effects of NAC on transcription factor expression in fetal hearts of diabetic offspring

**Figure 8:**
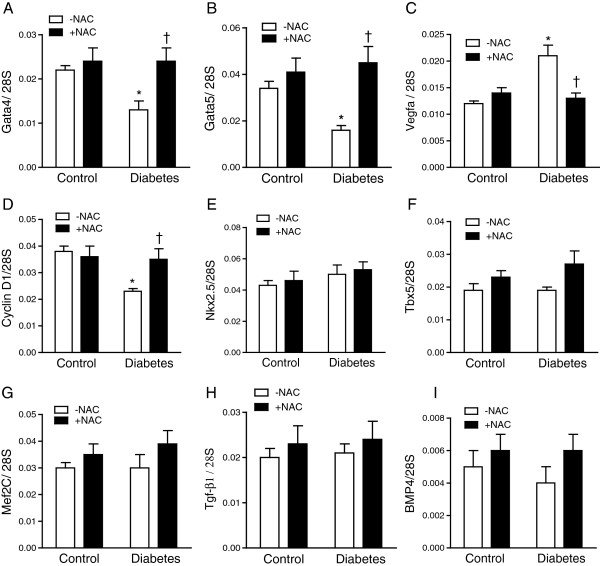
**Gene expression levels in fetal hearts at E11.5. (A-D)** The mRNA levels of *Gata4*, *Gata5*, *Vegf-a* and cyclin D1 were significantly altered in diabetic fetal hearts, which were restored to normal levels by NAC. **(E-I)** Neither pregestational maternal diabetes nor NAC treatment affected the mRNA levels of *Tbx5*, *Nkx2.5*, *Tgf-β1*, *Mef2c* and *Bmp4* in the embryonic hearts. Data are means ± SEM, *n* = 6-8 hearts per group. *P < 0.05 vs. untreated control, †*P* < 0.05 vs. untreated diabetes.

Pregestational diabetes alters gene expression levels in the developing heart [29]. To study the effect of pregestational diabetes and NAC treatment on genes essential for heart development, quantitative RT-PCR analysis was performed. Our data showed transcript levels of *Gata4* and *Gata5* were decreased in the fetal hearts of diabetic offspring at E11.5 (*P* < 0.05, Figure 8A-B). Since GATA4 and GATA5 regulate cell proliferation in the fetal heart, we evaluated expression levels of cyclin D1, an important cell cycle regulator. Our data showed that pregestational diabetes significantly decreased cyclin D1 mRNA levels in the fetal heart (*P* < 0.01, Figure 8D). On the contrary, *Vegf-a* mRNA levels were increased in the diabetic fetal hearts (P < 0.05, Figure 8C). These changes were all restored to control levels after NAC treatment (P < 0.05, Figure 8A-D). However, other cardiac transcription factors including *Nkx2.5*, *Mef2c* and *Tbx5* were not significantly altered by maternal diabetes or NAC treatment (Figure 8E-G). In addition, levels of *Bmp4* and *Tgf-β1*, which regulates cardiac valve formation, were not significantly altered (Figure 8H-I).

## Discussion

Pregestational diabetes is a major risk factor for CHDs in humans. However, the molecular mechanisms that lead to the development of CHDs and possible therapeutic approaches to prevent those defects are still not fully understood. It is generally believed that oxidative stress plays a major role in the induction of birth defects in diabetic fetus [12,16,30]. Here, we employed a mouse model of pregestational diabetes induced by STZ and studied the effects of NAC treatment on CHDs in the offspring of diabetic mice. Our data showed that pregestational diabetes resulted in a high incidence of CHDs and decreased cell proliferation associated with altered expression levels of *Gata4*, *Gata5* and *Vegf-a*. Importantly, GSH levels were decreased while ROS levels were increased in the fetal heart of pregestational diabetes. Notably, these abnormalities in the fetal heart were rescued by maternal treatment with NAC. Our study provides new evidence on the critical role of glutathione in embryonic heart development and suggests that NAC may have therapeutic potential in preventing CHDs in patients with pregestational diabetes.

To simulate congenital malformations induced by maternal diabetes without genetic modifications, several experimental approaches have been used, which include STZ- or alloxan-induced diabetes, and infusion of glucose to induce hyperglycemia. When diabetes or hyperglycemia is induced at the time of mating or within a few days after gestation, congenital defects in the central nervous system and skeletal malformation are observed in the offspring [31,32]. While congenital heart malformations have been observed in animal studies of diabetic pregnancy, a spectrum of defects that can arise has been less well characterized [33-35]. In the present study, diabetes was induced by STZ in female mice for at least one week before gestation. Our results show that pregestational diabetes induces embryopathy with a wide range of cardiovascular malformations including ASD, VSD, AVSD, TGA, DORV and TOF. These malformations of the cardiovascular system mirror congenital defects of neonates born to females with pregestational diabetes [6]. Thus, our model represents an appropriate animal model to study CHDs induced by pregestational diabetes.

Diabetes increases ROS production through increased activity of ROS generating enzymes and decreased activity of antioxidant enzymes [36,37]. Extensive evidence have shown the involvement of oxidative stress in diabetic embryopathy [38] and the importance of glutathione in regulating ROS levels and redox signaling [26]. In the present study, we demonstrated that ROS levels were significantly increased in the fetal heart of diabetic offspring. Furthermore, total glutathione, GSH and GSSG levels were decreased in the embryonic heart of diabetic offspring. To replenish GSH levels in the diabetic fetal heart, female mice with pregestational diabetes were treated with NAC, a precursor of cysteine essential for the production of GSH [18,39,40]. Notably, treatment with NAC increased GSH levels and decreased ROS levels in the diabetic fetal heart. Importantly, NAC treatment also significantly decreased CHDs induced by pregestational diabetes. These data suggest an important role of GSH depletion and excessive ROS production in the development of CHDs. Previous studies have shown that treatment with NAC *in vitro* or GSH ethyl ester *in vivo* reduces gross embryonic malformation induced by high glucose or maternal diabetes [10,16]. However, the beneficial effect of glutathione on cardiac development was limited to outflow tract defects induced by high glucose [20,34]. The present study further demonstrated the beneficial effects of NAC on a wide spectrum of cardiovascular malformations induced by pregestational diabetes *in vivo*. It should be noted that NAC treatment did not alter total glutathione levels in the fetal hearts of control mice. This is not surprising because intracellular GSH levels are regulated by a feedback inhibition to glutamate-cysteine ligase (GCL), a rate limiting enzyme in the production of GSH [41]. As such, the exogenous NAC participates in GSH synthesis only during oxidative stress conditions [39]. In addition, NAC also protects GSH from oxidation through its antioxidant properties independent of GSH synthesis [42], leading to an increased GSH/total glutathione ratio in both diabetic or control mice in the present study.

It is well documented that ROS regulates gene expression, cell proliferation and apoptosis [43]. In the present study, transcription factors that are critical to embryonic heart development including *Gata4*, *Gata5*[44,45] were downregulated in diabetic fetal hearts at E11.5. However similar to previous studies, *Vegf-a* mRNA levels were increased in diabetic fetal hearts [35]. Elevated expression levels of VEGF-A are associated with congenital heart defects [46,47]. High VEGF-A levels in fetal hearts inhibit epithelial-to-mesenchymal transition (EMT) in the endocardial cushion, which contributes to formation of atrioventricular septum [48,49]. Although a causal relationship between altered expression of *Gata4*, *Gata5*, *Vegf-a* and the development of CHDs in our study cannot be established, the fact that treatment with NAC restored their expression, improved cell proliferation via restoring cyclin D1 expression and prevented CHDs in diabetic offspring suggests that these transcription factors are sensitive to redox regulation and their alteration may contribute at least in part to cardiac malformation in pregestational diabetes.

Apoptosis is a highly regulated process and aberrant apoptosis may result in cardiovascular defects [50,51]. The present study showed that apoptosis in the endocardial cushion was increased in diabetic embryos. Additionally, treatment with NAC increased apoptosis and induced 10% septal defects in control embryos. These data are consistent with a role of apoptosis in cardiac malformation [51,52]. Cell proliferation was assessed using pHH3 staining, which marks cells undergoing mitosis. Notably, the number of pHH3^+^ cells in the endocardial cushion and myocardium was decreased in diabetic embryos, which was rescued by NAC treatment. Cell proliferation in endocardial cushion was also increased by NAC treatment in the control embryos. Since NAC treatment did not inhibit apoptosis in diabetic embryos in our study, increases in cell proliferation may represent a major effect of NAC in preventing CHDs in diabetes.

In conclusion, the present study demonstrated that pregestational diabetes induces a wide spectrum of CHDs similar to humans. Treatment with NAC increases GSH levels, decreases ROS levels in the fetal heart and prevents the development of CHDs in the offspring of pregestational diabetes. In women with pregestational diabetes, insulin is the primary treatment to achieve good glycemic control [53]. However, insulin treatment is not sufficient to decrease the risk of CHDs in the diabetic offspring to normal levels [38,54]. Even with optimal care and planning of diabetic pregnancies, the risk of CHDs in the offspring of diabetic mothers is not as low as in the offspring of nondiabetic mothers. Further studies are required to investigate whether NAC, an FDA approved drug either alone or in combination with insulin prevents CHDs in infants of women with pregestational diabetes.

## Abbreviations

ASD: Atrial septal defect; AVSD: Atrioventricular septal defects; Bmp4: Bone morphogenetic protein-4; CHD: Congenital heart defect; DHE: Dihydroethidium; DORV: Double outlet right ventricle; GSH: Reduced glutathione; GSSG: Oxidized glutathione; NAC: N-acetylcysteine; pHH3: Phospho-histon H3; ROS: Reactive oxygen species; STZ: Streptozotocin; TGA: Transposition of great arteries; Tgf-β1: Transforming growth factor-beta1; TOF: Tetralogy of Fallot; Vegf-a: Vascular endothelial growth factor-a; VSD: Ventricular septal defect.

## Competing interests

The authors declare that they have no competing interests.

## Authors’ contributions

HM designed the research, performed the experiments, analyzed data, and drafted and revised the manuscript. XL designed the research, performed the experiments, analyzed data, and revised the manuscript. NM, TJV, BLU, and LJW performed partial experiments. ACG analyzed heart morphology and revised manuscript. QF conceived and designed the research, interpreted the results of experiments, and revised the manuscript. HM, XL and QF are guarantors of this work and, as such, had full access to all the data in the study and take responsibility for the integrity of the data and the accuracy of the data analysis. All authors read and approved the final manuscript.
